# Multiaxial rotational loading compromises the transition zone of the intervertebral disc: Ex vivo study using next‐generation bioreactors

**DOI:** 10.1002/btm2.70033

**Published:** 2025-06-08

**Authors:** Amra Šećerović, Aapo Ristaniemi, Francesco Crivelli, Sarah Heub, Mauro Alini, Gilles Weder, Diane Ledroit, Stephen J. Ferguson, Sibylle Grad

**Affiliations:** ^1^ AO Research Institute Davos Davos Switzerland; ^2^ Swiss Center for Electronics and Microtechnology Alpnach Switzerland; ^3^ Swiss Center for Electronics and Microtechnology Neuchatel Switzerland; ^4^ ETH Zürich Institute for Biomechanics Zürich Switzerland

**Keywords:** bioreactors, intervertebral disc, multiaxial loading, transition zone, whole organ culture

## Abstract

Bioreactors have become indispensable tools in spine research, enabling long‐term intervertebral disc culture under controlled biological and mechanical conditions. Conventional systems are often limited to uniaxial loading, restricting their ability to replicate the complex, multidirectional biomechanics of the spine. To overcome this limitation, we developed a next‐generation bioreactor capable of simulating multiaxial motions while preserving the disc's biological environment. In this study, we investigated the effects of complex loading patterns on early disc degeneration by subjecting bovine whole‐organ discs to combined extension, lateral bending, and torsion at 0.3 Hz for 2 h daily over 14 days. To assess the impact of loading magnitude and the specific contribution of torsion, discs were exposed to either low‐ or high‐angle rotations, with or without torsional loading at higher angles. Histological analysis revealed a marked loss of glycosaminoglycans (GAG) and collagen type II within the inner annulus fibrosus and transitional nucleus pulposus (NP), encompassing the transition zone (TZ), as well as GAG depletion in the central NP. Matrix degradation was observed across all loading conditions, with the most severe breakdown occurring under high‐angle extension, bending, and torsion. All loading regimes induced cell death in the TZ and central NP, although torsion‐free loading better maintained cell viability. These findings highlight the TZ, alongside the commonly affected NP, as a critical early site of degeneration. The study further underscores the importance of incorporating multiaxial loading in disc degeneration models and provides new insights into the biomechanical mechanisms underlying disc pathology.

**Translational Impact Statement**

Whole organ models are indispensable for bridging the gap between in vitro tissue cultures and in vivo preclinical and clinical trials. Musculoskeletal tissues require the implementation of the complex mechanical environment. This study presents an exclusive organ model showcasing the intervertebral disc where mechanical forces in multiple degrees of freedom are applied and detrimental effects of combined rotations on the spine are highlighted. Our system advances opportunities for translational mechanobiological studies and preclinical evaluation of new treatments for musculoskeletal repair or regeneration.

## 1 INTRODUCTION

Intervertebral discs (IVDs) are essential components of the spine, responsible for distributing mechanical loads and enabling complex movements such as bending and twisting. Repetitive mechanical loading can predispose IVDs to microdamage, degeneration, and structural failure, ultimately compromising their integrity and contributing to functional impairments and chronic pain.^1^, ^2^ Advancing our understanding of the biomechanical mechanisms underlying disc pathology, alongside the development of effective therapeutic strategies, remains a key milestone in spine research.

Bioreactor systems have emerged as pivotal tools in this effort, offering controlled environments to simulate and study disc degeneration under physiological or pathological conditions.^3^, ^4^, ^5^, ^6^ These systems have proven particularly effective in applying dynamic or static uniaxial compressive loading to bovine whole organ disc models.^7^, ^8^ By manipulating loading parameters, bioreactor‐based studies have provided valuable insights into mechanically induced disc degeneration. For example, moderate cyclic compressive loading at low frequencies (0.1–0.2 Hz) generally maintains bovine discs in a near‐physiological state, while higher frequencies (5–10 Hz) tend to induce degenerative effects.^7^, ^9^, ^10^, ^11^ Although such models effectively replicate molecular, compositional, and structural hallmarks of degeneration,^9^ the specific site of tissue damage and the progression of regional changes remain unclear.

Traditionally, the nucleus pulposus (NP)—a centrally located, poro‐viscoelastic region—has been considered the primary site of disc degeneration. Its randomly organized extracellular matrix, rich in collagen type II and glycosaminoglycans (GAG), facilitates water retention and load distribution. These characteristics also make the NP susceptible to cell clustering, elevated catabolic activity, and matrix degradation.^12^ Emerging evidence, however, suggests a significant role of the annulus fibrosus (AF) in the degenerative process. Its highly organized lamellar structure, constituted of collagen type I, is subject to high strains that can compromise the structural integrity of the disc.^13^ Positioned at the interface between the AF and NP, the transition zone (TZ) has yet to be clearly defined or comprehensively characterized in the literature. Nonetheless, it is recognized to have a distinct structural and compositional profile,^14^ marked by a gradual transition in fiber organization—from aligned to disorganized—and in matrix composition, from predominantly collagen type I to a mixture of collagen types I and II, along with GAGs. This unique architecture suggests a potentially critical role for the TZ in maintaining the mechanical and structural coherence of the disc. However, the mechanical properties of the TZ have only recently been investigated in comparison to adjacent regions^14^, ^15^, ^16^ and incorporated into finite element models.^17^, ^18^

Bioreactor systems are well‐positioned to further elucidate the functional roles of these distinct disc regions in the degenerative processes. However, current systems are constrained by simplified loading protocols that fail to capture the multidirectional and complex mechanical environments of the human spine. While some studies using human or porcine disc models have incorporated more realistic spinal motions, they typically focus on biomechanical outcomes or inducing disc damage and herniation without controlling for biological variables like cellular viability or molecular responses.^19^, ^20^, ^21^

To overcome these limitations, bioreactors with two degrees of freedom have been introduced, enabling the application of combined loading modes such as compression and torsion. Increased torsional magnitudes were found to disrupt cellular metabolism,^22^ and low‐angle torsion with compression induced significant cell death in the NP and catabolic activation of matrix remodeling processes in the AF, although matrix composition remained unchanged.^23^ Additional studies investigating combined flexion and extension showed upregulation of catabolic markers in the AF,^24^ and reduced aggrecan levels in the AF at high‐angle flexion.^25^

**FIGURE 1:**
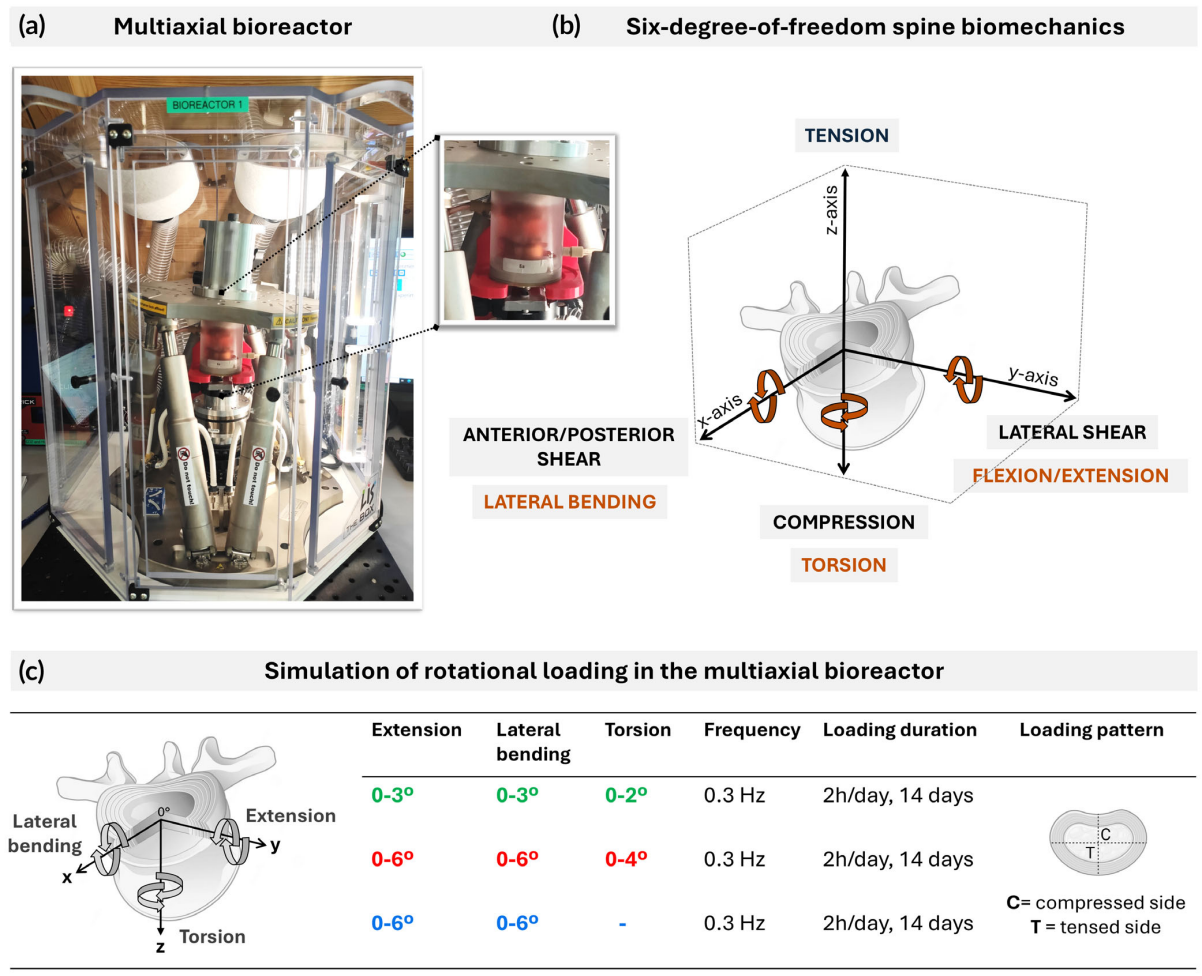
(a) Next‐generation multiaxial bioreactor designed for applying complex mechanical loading and precisely controlling the biological environment of bovine ex vivo whole organ models. The system comprises a hexapod actuator, control software, an incubator, and a previously described chamber with integrated mechanical interfaces and the specimen holder.^26^ A close‐up view of the chamber is shown. (b) Illustration of the six degrees of freedom in spine motion that can be simulated using the multiaxial bioreactor. (c) Experimental design and parameters for this study, testing combined rotational spine movements: extension and lateral bending, with or without the addition of torsion. The asymmetrical loading pattern indicates the directly loaded, compressed side of the disc and the counter‐loaded, tensed disc side.

To further investigate the effects of complex loading on early disc degeneration, we developed next‐generation multiaxial bioreactors (Figure 1a), capable of simulating six‐degrees‐of‐freedom spinal motions (Figure 1b) while ensuring the disc's sterility, viability, and precise environmental control. Key features of these multiaxial bioreactors include (1) a hexapod mechanical actuator for accurate simulation of multiaxial motions, (2) customized control software for precise control over loading parameters such as force, angular displacement, frequency, and duration, (3) an integrated incubator with temperature control, and (4) a specimen maintenance system, including a specialized chamber, mechanical interfaces, and a specimen holder to support a bovine whole organ model optimized for multiaxial loading.^26^

To design appropriate testing protocols, we conducted a comprehensive review of ex vivo and in vivo studies to identify spine movements commonly associated with disc health and degeneration. We focused on rotational movements typical in sports such as tennis, gymnastics, swimming, and contact sports. These motions often involve extension, bending, and twisting, which have been linked to radiological changes and back pain due to repeated stress and abnormal loading.^27^, ^28^, ^29^ Among these, torsion has been identified as particularly harmful to the AF, disrupting the lamellae,^30^ and significantly increasing herniation risk when combined with flexion.^31^ In our bioreactor setup, combined rotational loads were applied asymmetrically to simulate in vivo conditions where the loaded side of the disc experiences sustained compression, while the contralateral side undergoes tensile strain (Figure 1c). This asymmetrical loading paradigm is critical for modeling early degeneration, as it replicates high strains that are known to drive localized tissue failure and annular tears—even in the absence of degeneration.^32^

The primary objective of this study was to evaluate the effects of asymmetrical, multiaxial loading on early disc degeneration. A secondary aim was to determine the specific magnitudes of combined rotational motions that trigger degenerative changes. To address the latter, we developed and validated a finite element model tailored to the geometry, material properties, and internal structure of the bovine whole‐organ disc.^33^ This model captures the time‐dependent, dynamic behavior of discs under repetitive loading. From a simulation of 10 parameter combinations of the complex motion,^34^ two were selected for experimental validation in the bioreactor (Figure 1c).

We hypothesized that varying the magnitude of combined angular displacements would differentially affect the disc tissue. A motion involving 3° extension, 3° lateral bending, and 2° torsion was considered physiological and expected to maintain disc integrity and viability. In contrast, a higher‐angle combination at 6° extension, 6° lateral bending, and 4° torsion was predicted to induce structural and cellular changes indicative of early degeneration. To test this, we analyzed extracellular matrix composition and cell viability across key regions of the intervertebral disc: outer AF, inner AF, transitional NP, and central NP. The inner AF and transitional NP were defined to encompass the TZ, based on the observed gradual changes in fiber organization and matrix composition in bovine discs. Additionally, a torsion‐free high‐angle loading group was included to isolate the specific contribution of torsion in driving degeneration, further clarifying the role of individual mechanical loads in disc degeneration.

## 2 MATERIALS AND METHODS

### 2.1 ETHICS STATEMENT

Bovine IVDs from animals sacrificed for meat production were used for this study. No ethical approval was needed to carry out the experiments.

### 2.2 Bovine whole organ model

The whole organ IVD (diameter 20.1 ± 2.2 mm) model was prepared from 10 freshly slaughtered bovine tails from 7 male and 3 female cows (age 8.6 ± 2.4 months) following a previously established protocol for multiaxial loading.^26^ In brief, 7 mm of vertebral bone was retained above the cartilaginous endplates, and a milling machine (Proxxon, Germany) was used to create a cross‐shaped notch for fixation to the specimen holder and a central hole to facilitate nutrient diffusion. Throughout the preparation and milling process, the specimens were continuously cooled with Ringer's solution (Braun, Germany). Blood clots were removed using a jet lavage system (Pulsavac, Zimmer Biomet, USA), and the specimens were decontaminated for 12 min in 10% penicillin and streptomycin (Pen‐Strep)/phosphate‐buffered saline (PBS) solution (Life Technologies, USA) followed by 2 min in a 1% Pen‐Strep/PBS solution. Cleaned specimens were immersed in 30 mL of Dulbecco's Modified Eagle Medium (4.5 g/L glucose) supplemented with sodium bicarbonate, pyruvate, 1% Pen‐Strep, 2% foetal calf serum (FCS; #35‐010‐CV, Corning, CA, USA), 1% ITS+ (Corning), 1% non‐essential amino acids (Life Technologies), 25 mmol/L HEPES (Life Technologies), 50 μg/mL ascorbate‐2‐phosphate, and 50 μg/mL primocin (InvivoGen, USA). The specimens were kept in free‐swelling conditions until loading commenced the following day. Each loading group consisted of four randomly assigned samples sourced from 3 or 4 different tails. Non‐loaded control samples were collected from each tail and maintained under free‐swelling conditions until processing on the second day after isolation (referred to as day 2 controls). These samples were used as reference samples for histology and immunohistochemistry. As reference samples for relative quantification of changes in gene expression, additional non‐loaded control discs were prepared from 3 separate tails from 2 male and 1 female cows (age 10.7 ± 1.9 months, diameter 23.7 ± 1.6 mm) and processed immediately after isolation (referred to as day 0 controls).

### 2.3 Multiaxial loading protocol

Disc specimens were assembled under sterile conditions using a specimen holder secured with top and side screws, mechanical interfaces, and chamber setup as previously described.^26^ During assembly, the specimens were intermittently hydrated with the culture medium. Once assembled, chambers were filled with 50–58 mL of medium to completely submerge specimens and their holders. Chambers were then mounted in the multiaxial bioreactor and subjected to one of three loading protocols combining rotational movements: (group 1) Extension 0–3°, Lateral bending 0–3°, and Torsion 0–2°; (group 2) Extension 0–6°, Lateral bending 0–6°, and Torsion 0–4°; (group 3) Extension 0–6° and Lateral bending 0–6° (Figure 1c). Each group underwent independent experimental runs, with daily loading sessions of 2 h at 0.3 Hz over a total of 14 days. To simulate the combined rotations as in the disc in vivo, the sinusoidal loading was applied in phase and phase‐adjusted to target approximately one‐quarter of the disc area, focusing the loading stress on a specific corner of the disc (Figure 1c). Following the loading sessions, chambers were transferred to an incubator maintained at 37°C, 5% CO_2_, and 85% humidity. Throughout the loading phase in the bioreactor and free‐swelling recovery phase in the incubator, specimens were maintained in the culture medium at 37°C. During the recovery period, humidity, CO_2_ levels, and gas exchange were controlled by replacing the chamber lid opening with a sterile 0.22 μm filter. The culture medium was changed every 3–4 days to maintain optimal disc nutrition and pH, with the volume noted at each change.

### 2.4 Measurements and harvesting

Disc height changes were measured daily after loading and free‐swelling recovery using a custom‐made laser system. This method detects the position of the mechanical interface on the external part of the chamber, which is in contact with the disc inside the chamber. Height change after loading was calculated as the difference between the heights measured before and after loading on the same day. Height change after swelling was calculated as the difference between the height before loading and the height recorded before loading on the previous day. Relative IVD height change was expressed as a function of disc height manually measured during tissue processing on the final day. Height was measured from one side of the cartilaginous endplate to the other at the deepest point of its concave surface.

Media samples were collected daily after loading and swelling using a 1 mL syringe (B.Braun, Melsungen, Germany) through an opening in the lower part of the chamber. For GAG analysis, 0.5 mL of medium was immediately stored at −20°C. For ELISA, 0.5 mL of medium was snap‐frozen and stored at −70°C. Disc tissue samples were collected at the 15‐day endpoint after the final overnight recovery. During chamber disassembly, the loaded side of the specimen was carefully identified and marked with a skin marker. Each specimen was longitudinally bisected through the loaded area, ensuring that both halves contained the directly loaded (compressed) side and counter (tensed) side of the disc. One sagittal half was further transversally sectioned through the disc center, and the resulting two pieces were snap‐frozen in liquid nitrogen for histological analysis. The second sagittal half was cleaned of bone by cutting close to the cartilaginous endplate. The remaining soft tissue was separated into loaded and counter regions of the outer AF. Between 100 and 200 mg of tissue from each region was immediately processed for RNA isolation.

### 2.5 RNA isolation and RT‐qPCR

Outer AF tissues were finely chopped using a blade and enzymatically digested for 1 h at 37°C in 2 mg/mL pronase (Roche, Switzerland) prepared in a basal medium. The digestion was halted by adding 500 μL of FCS. The digested tissues were washed with PBS, snap‐frozen in liquid nitrogen, and homogenized using a custom‐made hammering tool.^35^ The homogenate was vortexed with 1 mL TRI‐Reagent (Molecular Research Center, USA) and 5 μL of Poly‐Acryl‐Carrier (Molecular Research Center). Further tissue disintegration was achieved by processing the samples with an 8 mm stainless steel bead in a tissue lyser (Retsch, Germany) for three 3‐min cycles at 25 Hz. The resulting lysate was mixed with 100 μL of 1‐bromo‐3‐chloropropane (Sigma‐Aldrich, Merck, Germany) and incubated on an orbital shaker for 15 min. After centrifugation, the upper aqueous phase containing RNA was carefully collected and purified using the RNeasy MINI Kit (Qiagen, Germany), according to the manufacturer's instructions. Reverse transcription was performed using the Vilo Superscript Kit (Thermo Fisher, USA) in a 20 μL reaction volume containing 400 ng of RNA. The resulting cDNA was diluted in TE buffer (Promega, Switzerland) to a final concentration of 4 ng/μL. Real‐time quantitative polymerase chain reaction (qPCR) was conducted using a TaqMan Universal Master Mix (Applied Biosystems, USA) with a total reaction volume of 10 μL. The following target bovine genes were analyzed using custom‐designed primers (Supp. Table 1): matrix metallopeptidases (MMP1, MMP3, MMP9 and MMP13), a disintegrin and metalloproteinase with thrombospondin motifs (ADAMTS4, ADAMTS5), interleukins (IL‐1*β*, IL‐6), tumor necrosis factor‐alpha (TNF‐*α*), collagen types I and II (COL1, COL2), aggrecan (ACAN) and cartilage oligomeric matrix protein (COMP). For additional genes, including ribosomal protein lateral stalk subunit P0 (RPLP0), MMP19, IL‐8, and elastin (ELN), a gene expression assay (Life Technologies, USA) was used (Supp. Table 1). All reactions were run as duplicates and performed using the QuantStudio 7 instrument (Thermo Fisher, USA) under standard conditions. Relative gene expression was calculated using the comparative *Ct* method (ΔΔ*Ct*), with RPLP0 as the endogenous control and the average gene expression value of day 0 non‐loaded samples as the reference.

### 2.6 Histological analysis

Frozen tissues were transversely sectioned into consecutive 10 μm‐thick slices using a cryotome (Thermo Fisher Scientific, USA). Sections were either used unfixed for cell viability assessment or fixed in 100% methanol for 10 min prior to general staining methods or in methanol and 3% hydrogen peroxide (Sigma‐Aldrich, Merck, Germany) for immunohistochemistry.

Cell viability was evaluated using the lactate dehydrogenase and ethidium homodimer‐1 method.^26^ The number of viable (blue and blue/orange) and dead (orange) cells was quantified using the ImageJ program in four random regions of interest in the outer AF, inner AF, and transitional NP, and six in central NP. Cell viability was calculated as the ratio of viable cells to the total cell count. For GAG visualization, sections were stained for 10 min with Weigert's hematoxylin (Sigma‐Aldrich), followed by 0.1% fast green for 6 min and 0.1% safranin‐O (Sigma‐Aldrich) for 12 min. Sections were differentiated in ethanol and mounted with Eukitt medium (Sigma‐Aldrich). To identify lamellar structures, sections were stained with Weigert's hematoxylin for 8 min and picrosirius red (Sigma‐Aldrich) for 1 h, washed in acidified water, dehydrated in ethanol, and mounted.

For collagen type I and II detection, sections were treated with hyaluronidase at 1 U/mL (Sigma‐Aldrich, #H3506) for 30 min at 37°C, blocked with 1:20 normal horse serum (Vector Laboratories, USA) for 1 h at room temperature (RT), and incubated with primary antibodies against collagen type I (Sigma‐Aldrich, #C2456, diluted 1:2000 with PBS‐T) or collagen type II (CIICI, DSHB, diluted to 2 μg/mL IgG with PBS‐T) for 30 min at RT. This was followed by incubation at RT with an anti‐mouse IgG secondary antibody (Vector Laboratories) for 30 min, ABC complex (Vector Laboratories) for 30 min, and color developed with DAB solution for 4 min. Sections were counterstained with Mayer's hematoxylin (Sigma‐Aldrich) for 20 s, dehydrated in ethanol, and mounted.

Images were captured using an Olympus microscope (Tokyo, Japan) under transmitted, fluorescence, or polarized light. GAG and collagen type I and II‐positive regions were quantified on consecutively stained tissue sections using the ImageJ program. Picrosirius red‐stained sections were used to delineate the AF at three representative points based on its characteristic lamellar structure (Figure 3a). These indications were mirrored onto adjacent sections stained for collagen type I, collagen type II, and GAG. The AF region was outlined using a freehand selection tool and subsequently divided to differentiate the outer and inner AF regions. The average width of the inner AF in each specimen was used to define the adjacent transitional NP immediately beyond the lamellar structure, while the remaining NP tissue was designated as central NP. The inner AF and transitional NP were defined to encompass the TZ based on the observed gradual changes in fiber organization and matrix composition. For each defined region, the percentage of positively stained area was quantified. As GAG and collagen type II are typically absent in the outer AF, the minimal signal detected (0.1% ± 0.1% and 1.3% ± 0.8%, respectively) was considered a technical artifact and excluded from graphical representation. Tissue cracks within the inner AF were assessed on safranin‐O/fast green‐stained sections by quantifying the non‐stained tissue background, representing voids or discontinuities in the tissue matrix. For each loaded sample, analyses were conducted on sections from two adjacent locations at the disc center, sectioned from each disc half. Data was presented as individual values from both sites, except when image quality allowed for quantification at only one site. Non‐loaded control samples of sufficient quality for quantification were analyzed on sections originating from one disc half.

### 2.7 Medium analysis

Non‐diluted media samples were analyzed in duplicates for IL‐6, while GAG was measured in triplicates after dilution. GAG release was quantified using a direct spectrophotometric microassay, using 1,9‐dimethyl‐methylene blue color reagent with chondroitin 4‐sulfate sodium salt from the bovine trachea (a mixture of isomers from Merck, Germany) as a standard. The release of IL‐6 was quantified using specific bovine ELISA kits (Kingfisher Biotech, USA; catalogue number DIY0670B‐004) according to the manufacturer's instructions.

Absorbance values were measured using a plate reader (Tecan Trading AG, Austria) at 535 nm for GAG and at 550 nm for IL‐6. The amounts of GAG and interleukins in the medium were established based on the measured concentration and the medium volume in the chamber at each specific time point. For each sample, the release values were normalized to the disc volume, derived from manual measurements of disc diameter and height (excluding bony parts) during tissue processing on the initial and final day, respectively, assuming a regular cylindrical shape. Total cumulative releases were calculated from the first medium change by summing the daily release values after loading and free‐swelling periods and shown on the graph as release values recorded after each period of 24 h that involved loading and swelling.

### 2.8 Statistical analysis

Statistical analysis was performed using GraphPad Prism 9 software (GraphPad, USA). Before any analysis, data normality was evaluated using the Shapiro–Wilk test. For comparison between two groups, an unpaired parametric *t*‐test was applied to data with normal distribution and comparable standard deviations, while the unpaired non‐parametric Kolmogorov–Smirnov test was used for non‐normally distributed data. Three or more loading groups were assessed using one‐way ANOVA with Tukey's multiple comparisons test or non‐parametric Kruskal–Wallis test with Dunn's multiple comparisons test. To evaluate the combined effects of loading conditions and time, a repeated measures two‐way ANOVA with the Geisser–Greenhouse correction and Tukey's multiple comparisons test was conducted. Statistical significance was defined as a *p*‐value less than 0.05.

## 3 RESULTS

### 3.1 Minimal disc height changes

**FIGURE 2:**
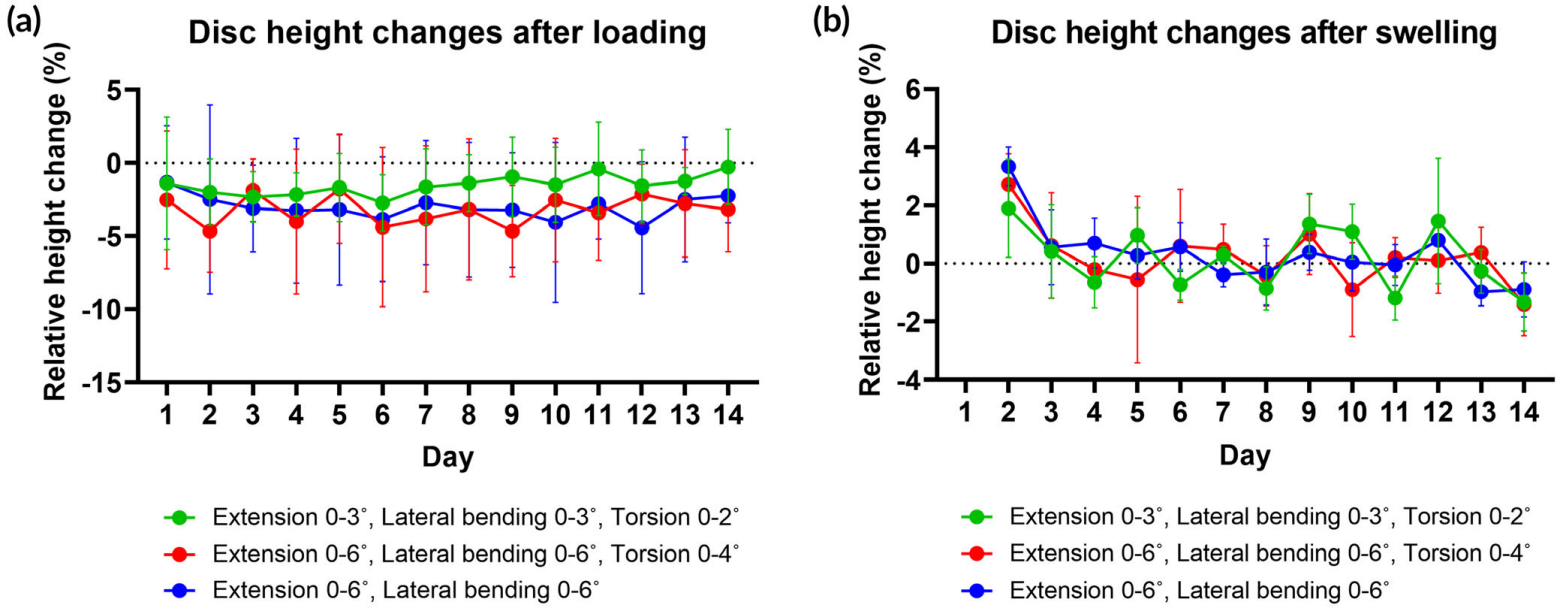
Disc height changes were measured daily over 14 days, following loading and free‐swelling recovery. Measurements of height after swelling were first recorded on day 2, after the recovery from initial loading on day 1. Changes in height following loading were calculated relative to the pre‐loading height recorded on the same day. Changes in height following swelling were calculated relative to the height before loading recorded the previous day. Data show mean values from 4 samples and standard deviation. A repeated measures two‐way ANOVA test was performed to analyze the effects of loading parameters and time on height changes.

Discs lost 2.6% ± 3.4% of their height daily following loading, with no significant differences observed between groups (Figure 2a). The group subjected to lower angles of extension/bending/torsion showed, in the second week of loading, a trend toward less shrinkage compared to the group loaded at higher angles of extension/bending/torsion and extension/bending. Discs from all groups demonstrated complete height restoration after overnight recovery and maintained a consistent recovery height throughout the experiment (Figure 2b).

### 3.2 Regional extracellular matrix loss

**FIGURE 3:**
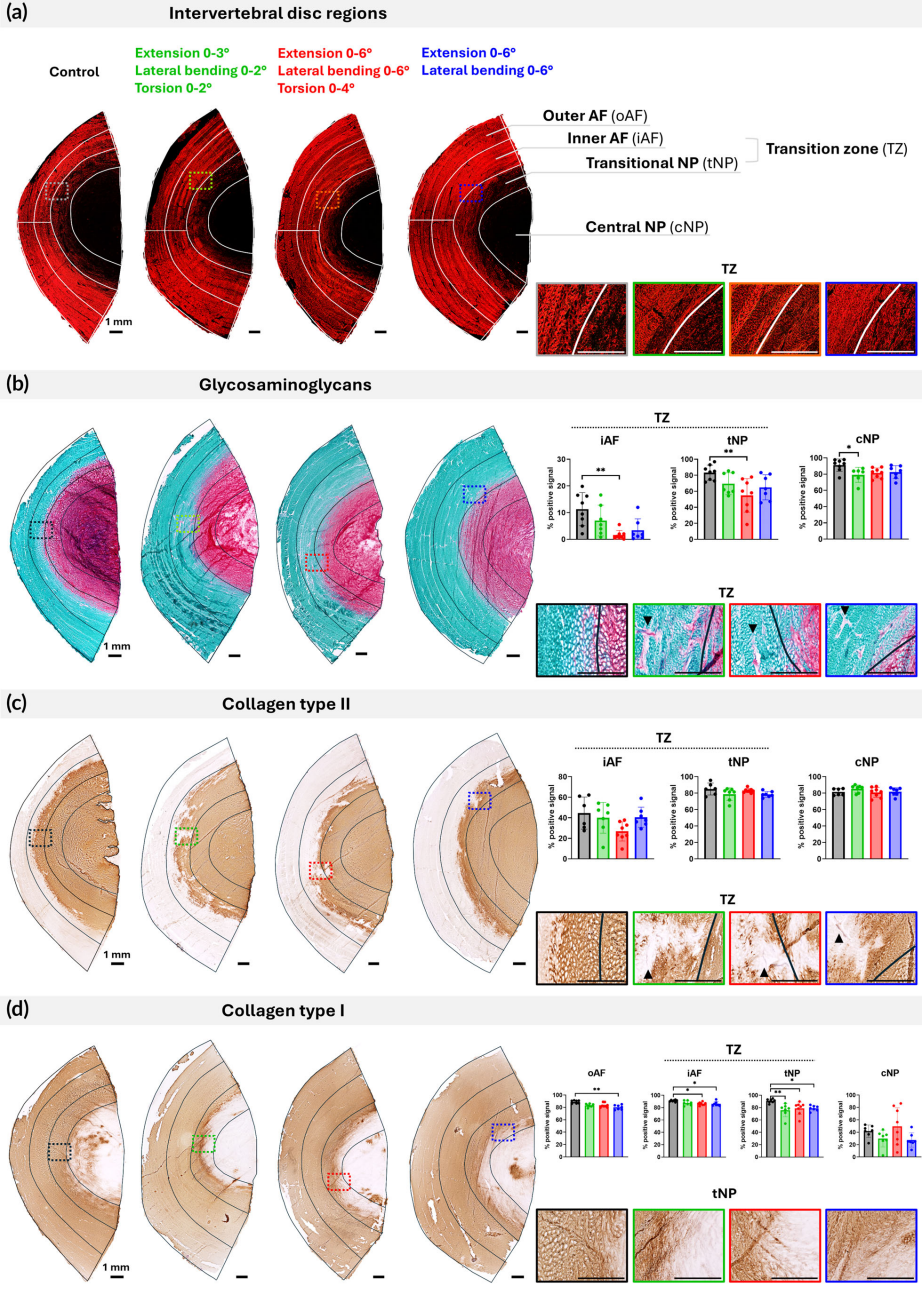
Analysis of the extracellular matrix in different regions of the intervertebral disc. Panels (a)–(d) show representative macroscopic and close‐up images of a control and a loaded specimen with altered composition from each experimental group, analyzed using different staining methods. Graphs (b)–(d) show the analysis of positive signals from all control and loaded specimens. For loaded groups, data points represent individual measurements obtained from two adjacent locations at the disc center for each sample, except when image quality permitted quantification at only one site. (a) Picrosirius red staining highlights the organized lamellae defining the outer and inner annulus fibrosus (AF), and the randomly organized nucleus pulposus (NP), which includes transitional and central regions. The inner AF and transitional NP encompass the transition zone (TZ). (b) Safranin‐O staining visualizes glycosaminoglycan (GAG) content in red, while fast green counterstains fibrous components. (c), (d) Immunolabeling identifies the distribution of collagen type I and type II. Close‐up images show a loss of GAG and collagen type II signals in the TZ. Arrows indicate vertical tissue cracks within this region, with cells aligned along the cracks (upward arrows) and GAG deposition alongside cells (downward arrows). Scale bars indicate 1 mm. Statistical analysis between all groups was performed using one‐way ANOVA or Kruskal–Wallis test, where *p* < 0.05 (*) and *p* < 0.01 (**) were statistically significant.

Qualitative histological analysis revealed extracellular matrix changes in multiple disc regions, with at least two specimens per experimental group showing detectable loss of GAG or collagen type II within the TZ (Figure 3b, c). GAG loss was evident as diminished or absent red staining, though it was visually less pronounced than collagen type II depletion, considering collagen type II prevalence in the inner AF. Collagen type II degradation was characterized by irregularly shaped, discolored tissue patches, in contrast to the homogeneous matrix structure observed in the controls.

Quantitative analysis confirmed a significant reduction in matrix components in discs subjected to higher angles of extension/bending/torsion compared to controls. This reduction was particularly evident for GAG in the inner AF and transitional NP, encompassing the TZ (Figure 3b). A notable trend toward collagen type II loss was also observed in the inner AF of this group (Figure 3c). Similarly, the high‐angle extension/bending group showed a strong trend toward GAG reduction in the inner AF, comparable to the levels in the significantly affected group. All loading groups showed a general trend toward GAG depletion in the central NP, with the low‐angle extension/bending/torsion group displaying a significant reduction compared to controls. In some inner AF regions, matrix loss coincided with tissue cracks, where cells were seen aligning along the fissures and depositing GAG (Figure 3b; arrowhead). However, quantification of tissue voids revealed no significant differences between control (5.5% ± 2%) and experimental groups: low (5.9% ± 2.2%) and high‐angle extension/bending/torsion (5.4% ± 1.6%) and high‐angle extension/bending (6% ± 1.7%).

In the central NP, at least two samples per group displayed cell clustering, in contrast to the typically isolated, single‐cell distribution seen in control samples (Supp. Figure 1). While annular lamellar fiber structure remained mostly intact across specimens (Figure 3a), one disc from the high‐angle extension/bending/torsion group exhibited disorganized annular lamellae in the TZ (Supp. Figure 2A), accompanied by a wavy pattern of GAG and collagen type II staining (Supp. Figure 2B, C). Notably, this specimen also showed abnormally elevated collagen type I levels in the NP (Supp. Figure 2D).

Collagen type I levels were mildly reduced across the outer and inner AF in all groups, with some of these differences reaching statistical significance compared to controls (Figure 3d). The transitional NP exhibited the most consistent and pronounced collagen type I loss. In the central NP, both the low‐angle extension/bending/torsion and high‐angle extension/bending groups showed a trend toward reduced collagen type I, while levels in the high‐angle extension/bending/torsion group remained on average similar to controls, likely due to high inter‐sample variability.

### 3.3 GAG and interleukin‐6 release in the medium

**FIGURE 4:**
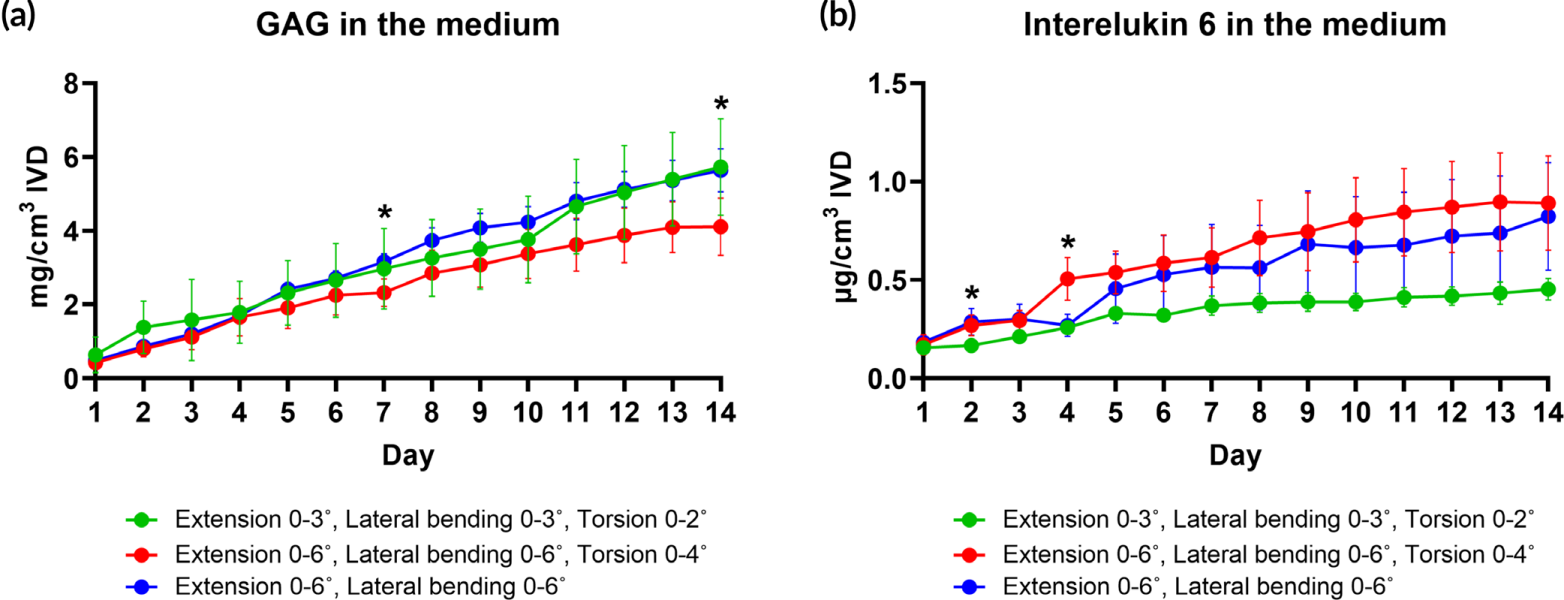
Absolute release of glycosaminoglycans (GAG; a) and interleukin‐6 (IL‐6; b) until the first medium change at day 4 (green and red groups) and day 5 (blue group), followed by cumulative release until day 14 of daily loading and free‐swelling periods. Data are presented as mean values from 4 samples and standard deviation. A repeated measures two‐way ANOVA test was performed to analyze the effects of loading parameters and time, where a *p* < 0.05 (*) was statistically significant between the red and blue groups (a), red and green groups on day 2 (b) and red and green and red and blue groups on day 4 (b).

The daily release of GAG into the medium following loading and swelling was consistent across all loaded groups (Figure 4a). A day‐by‐day analysis revealed that the group subjected to higher angles of extension/bending/torsion showed, on days 7 and 14, a significantly lower amount of GAG released compared to the group loaded with extension/bending only. This divergence aligned with the overall trends in slope changes observed for these groups, beginning on day 5, where the former exhibited a decreasing trend and the latter an increasing trend.

Daily IL‐6 release into the medium varied between groups (Figure 4b). The group loaded at higher angles of extension/bending/torsion exhibited significantly elevated IL‐6 levels on day 2 compared to the lower‐angle group, and on day 4 compared to both the lower‐angle extension/bending/torsion group and the higher‐angle extension/bending group. Over time, the higher‐angle groups demonstrated an increasing trend in IL‐6 release, whereas the lower‐angle extension/bending/torsion group released minimal IL‐6 levels from day 5.

### 3.4 Region‐specific effect on cell viability

**FIGURE 5:**
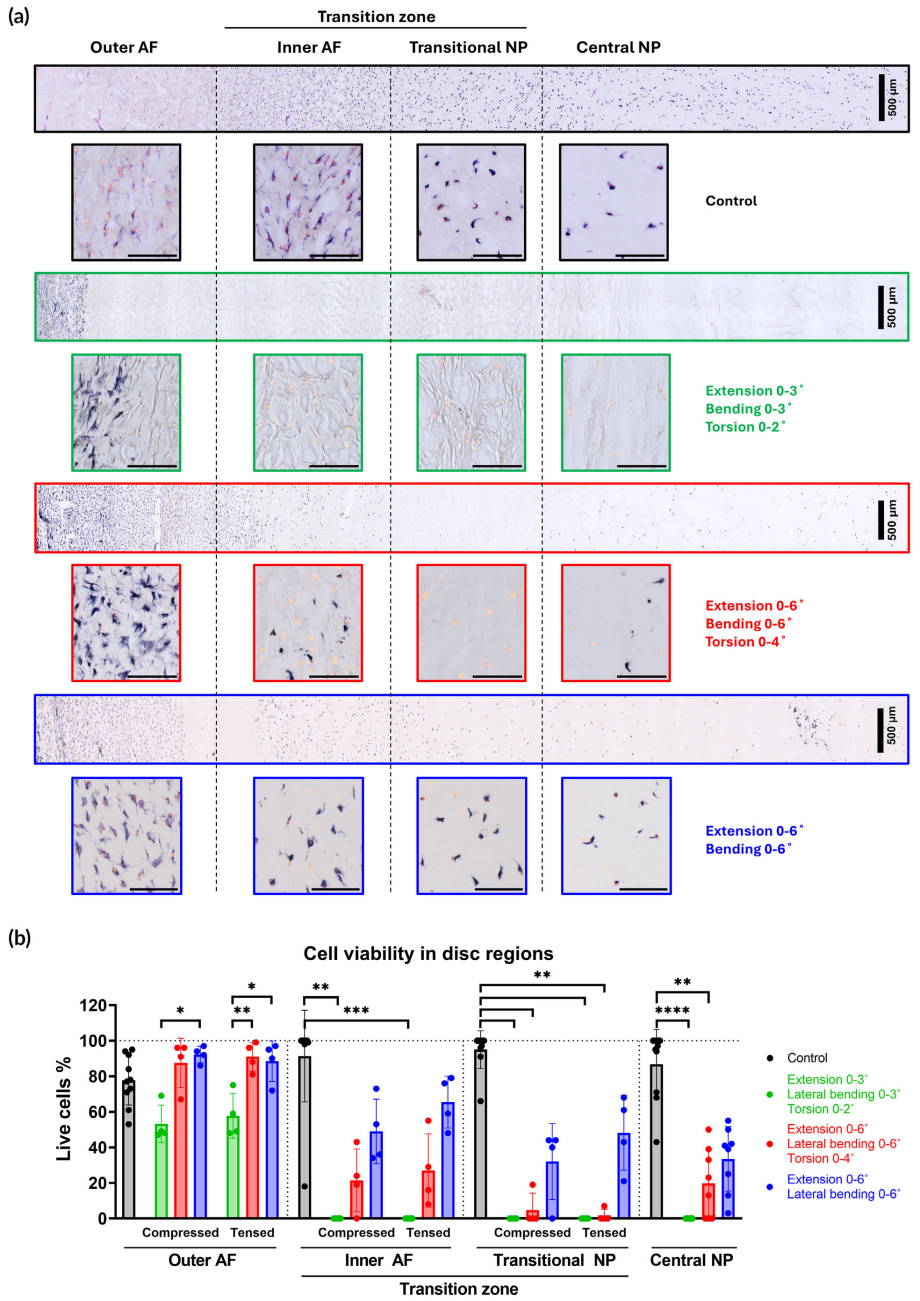
Cell viability was visualized and quantified in sections from non‐loaded controls and groups subjected to different loading parameters, stained with lactate dehydrogenase and ethidium homodimer. (a) Representative images show live cells (blue and blue/orange) and dead cells (orange) across a portion of the disc, encompassing the outer and inner annulus fibrosus (AF) and the transitional and central nucleus pulposus (NP). Higher‐magnification images highlight selected regions of interest (scale bar 100 μm). (b) Quantification of cell viability in the regions of interest, including from the directly loaded side in compression and the counter‐loaded side in tension. Data represent the mean ± standard deviation of four loaded samples per group and ten control samples. Central NP data were derived from both counter‐loaded and directly loaded disc regions within the same section. Statistical analysis between loaded and counter‐loaded regions within individual groups was performed using parametric or non‐parametric *t*‐test. Different groups within the same region were analyzed using one‐way ANOVA or the Kruskal–Wallis test, where *p* < 0.05 (*), *p* < 0.01 (**), *p* < 0.001 (***) and *p* < 0.0001 (****) were statistically significant.

Cell viability gradually declined from the outermost to innermost regions of discs across all experimental groups, with cells located in the TZ and central NP being particularly affected by cell death (Figure 5a, b). In the group exposed to lower angles of flexion/bending/torsion, cell viability in the outer AF was significantly reduced by 50%, whereas in the groups subjected to higher angles of flexion/bending/torsion and flexion/bending, viability levels were maintained similar to controls. Non‐loaded controls exhibited lower metabolic activity in the outer AF, leading to slightly reduced viability and brighter staining compared to mechanically stimulated specimens. However, most controls retained high viability and intense staining in the inner AF and NP.

In deeper regions, all loaded groups showed reduced viability compared to controls. The low‐angle group exhibited nearly zero viability in the TZ and central NP, while the high‐angle flexion/bending/torsion group had similar viability loss in the transitional NP. The high‐angle extension/bending group demonstrated greater cell survival rates in the TZ and central NP than the other two loaded groups. Across all groups, cell viability remained similar between the compressed and tensed sides of the AF and transitional NP.

### 3.5 Gene expression

**FIGURE 6:**
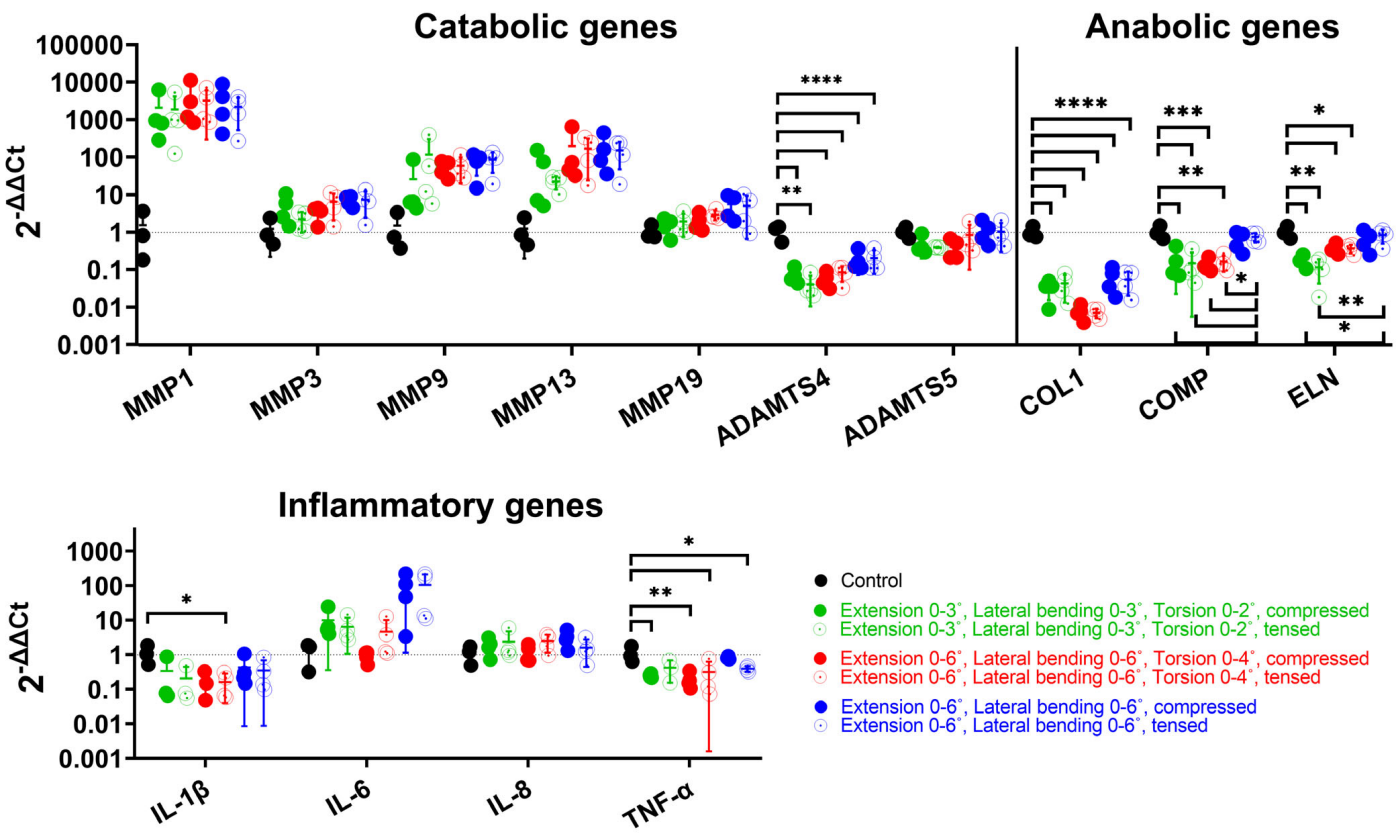
Expression of catabolic, anabolic, and inflammatory genes in the outer annulus fibrosus loaded in compression or tension and control samples. Gene expression was quantified using the comparative *Ct* method (ΔΔ*Ct*) normalized to an endogenous control gene and day 0 control samples. Samples below the detection limit were not shown on the graph. Statistical analysis between loaded groups, controls, and loaded groups, and compressed and tensed regions of individual groups was performed using the one‐way ANOVA or Kruskal–Wallis tests, where *p* < 0.05 (*), *p* < 0.01 (**), *p* < 0.001 (***) and *p* < 0.0001 (****) were statistically significant.

Gene expression analysis of the outer AF revealed increased expression of matrix‐degrading enzymes in loaded groups compared to controls, with MMP1 showing the highest upregulation, followed by moderate increases in MMP9 and MMP13, and a slight increase in MMP3. Despite an average 3700‐fold increase in MMP1 compared to control discs, these differences were not statistically significant. MMP19 expression remained similar to controls. No differences in MMP expression were recorded between groups (Figure 6). In contrast, ADAMTS4, a member of the catabolic enzyme family, was significantly downregulated in all groups relative to controls, while ADAMTS5 remained unchanged across all conditions.

COL1 was significantly downregulated in all groups compared to controls. Other anabolic genes, such as COMP and ELN, were downregulated in the low‐ and high‐angle extension/bending/torsion but remained comparable to controls in the flexion/lateral bending group. Notably, the counter (tensed) region of the latter group exhibited a significant difference in COMP expression compared to the other two groups and ELN expression compared to the low‐angle extension/bending/torsion group.

Expression of inflammatory genes varied slightly among the groups. IL‐6 showed upregulation in some groups, particularly in the high‐angle extension/bending group, although the differences were not significant. IL‐8 expression remained comparable to controls, while IL‐1*β* was generally maintained similar to controls or downregulated in the high‐angle extension/bending/torsion group. TNF‐*α* was downregulated in all groups in at least one region of the outer AF compared to controls. Across all loading conditions, gene expression changes were consistent within the AF, regardless of whether it was directly loaded in compression or counter‐loaded in tension.

## 4 DISCUSSION

The innovative experimental approach using next‐generation bioreactors offers new insights into the intricate relationship between complex spinal motions and disc degeneration. Notably, the study highlights the susceptibility of specific disc regions to matrix breakdown when subjected to combined rotational loading. The TZ, particularly within the lamellar AF, emerges as the primary site of GAG and collagen type II depletion, reinforcing the hypothesis that the AF is the mechanically weakest region of the disc and likely the initial site of degeneration.^36^ GAG loss was additionally observed in the central NP region, suggesting a critical role of inner disc regions in the response to mechanical strains induced by combined rotations. Furthermore, this study underscores the necessity of incorporating complex motions in bioreactor experiments, as prior ex vivo and long‐term in vivo studies, which focused solely on compression, did not identify such compositional changes in the TZ.^37^

The observed matrix breakdown may be attributed to high shear stresses, similar to those previously identified in lateral bending and flexion.^38^ High intradiscal pressures in the inner AF under asymmetrical torques^39^ further support this mechanism, as such pressures have been shown to reduce lamellar adhesion strength and induce fractures.^40^ In the present study, microfractures were observed adjacent to regions of matrix loss. However, due to their small size and the presence of GAG deposits within the tissue voids, it was challenging to demonstrate quantitative differences between the groups. Radiological assessment techniques may provide a more effective approach to investigating the relationship between matrix degradation and structural damage. Positive GAG staining within cracks in the inner AF has also been previously reported in 3‐month‐bent mouse discs.^41^ The authors attributed this effect to GAG deposition resulting from either compression‐guided differentiation of fibroblasts into chondrocyte‐like cells or active cell migration. Our findings align with this hypothesis, suggesting that localized GAG accumulation within microcracks may represent a mechanism of tissue repair. Although matrix loss and cracking in this study were not accompanied by herniation, previous biomechanical studies have shown that torsion and flexion can increase herniation risk, particularly at the junction of the endplate and AF.^31^ Finite element studies have also demonstrated that fiber strain in the inner AF, induced by combined lateral bending and torsion or flexion and torsion, can initiate ruptures that propagate toward the outer AF.^42^, ^43^ In this study, the outermost disc regions appeared to be the most resilient to rotational loading, compared to other disc regions, exhibiting higher cell viability and minimal structural and compositional alterations. Nonetheless, cracks were observed in the outer AF in several samples, indicating that even this region may be vulnerable under certain conditions. We suggest further investigating the progression toward structural failure with prolonged loading durations or an increased number of loading cycles.

Under combined rotational loading, discs exhibited an average height loss of only 2.6%, likely due to the absence of direct axial compression. In contrast, typical bioreactor studies applying compression have reported 10%–20% height loss attributed to NP shrinkage from water loss.^5^ Such compressive stress can strain the inner AF, accelerating cell death in this region during a long‐term disc culture.^26^ Given the minimal axial compressive loading in this study, the significant cell death and matrix depletion in the TZ are more likely attributed to alterations in axial and radial strains. These strain patterns have previously been shown to occur under complex loading, both in physiological conditions and degeneration.^32^ Notably, the same study identified a relationship between subtle disc height reductions and reduced NP pressure, which in turn increases radial tensile stress and places the inner AF at risk of damage. This finding suggests a potential mechanism underlying the matrix breakdown observed in this study. Nevertheless, it remains unclear whether the changes in the inner AF weaken the transitional NP region and allow the propagation of stresses and cellular death toward the disc center or whether the changes in both regions occur simultaneously.

Matrix breakdown and cell death developed even at low loading frequencies of 0.3 Hz, challenging the conventional view that such frequencies are beneficial to disc health.^44^ Instead, our results suggest that even low‐frequency complex mechanical loading can have detrimental effects, particularly with sufficient loading cycles. This is further supported by another study showing that low‐frequency (0.5 Hz) cyclic loading and flexion can damage the inner AF, with more severe changes occurring at higher cycle counts.^45^ However, considering that even the low‐angle loading significantly impacted the disc, the effect of loading frequency on degeneration should be further investigated, particularly in relation to the spatial distribution of mechanical stress.

Following established links between mechanical stresses and angles of rotation,^46^ and the predictions of our computational model,^34^ combined rotations at higher angles (4° and 6°) had a more pronounced degenerative effect on the matrix breakdown than lower angles (2° and 3°). However, contrary to our initial hypothesis that lower angles would preserve the disc in a physiological state, a significant cell death was observed in parts of the outer AF, the inner AF as well as the transitional and central NP, potentially due to altered stress distributions. Alternatively, the asymmetrical complex loading at lower magnitudes may have been insufficient to adequately stimulate nutrient flow. Symmetrical loading should be, therefore, taken into consideration when designing protocols for a physiological loading group. Prior research also showed a combination of compression with torsion at only 2° reduced NP cell viability significantly and to a greater extent than when these loads were applied individually.^23^ Considering that the outcomes of this study do not suggest a correlation between cellular death and matrix breakdown, we hypothesize that cellular damage may precede or differ from matrix degradation in the initiation of degeneration. To investigate this, analyzing samples at earlier time points is needed to investigate potential differences between cellular and extracellular matrix responses to complex mechanical loading and their dependence on the applied angles.

The matrix breakdown may be driven by metalloproteinases— particularly MMP1 with an average 3700‐fold increase— whose gene upregulation in the outer AF in this study is consistent with earlier research examining the effects of flexion/extension^24^, ^25^ and torsion with or without compression.^22^, ^23^ Despite a high fold increase compared to controls, the differences did not reach statistical significance, likely due to the unequal sample sizes of control and loaded groups and the sample variability. In contrast, ADAMTS genes encoding aggrecanases were found downregulated or unchanged, suggesting their minimal role in the degradation process. This finding is supported by earlier research showing a shift from aggrecanase‐driven degradation (observed in healthy discs) to metalloproteinase‐mediated cleavage of aggrecan in response to compressive overloading.^47^ However, considering that the expression of MMPs was similarly upregulated across different loading protocols which exhibited differential effects on the matrix, it remains unclear whether the matrix breakdown primarily resulted from enzymatic activity or was directly caused by mechanical disruption. Future studies should focus on the spatiotemporal correlation analysis^48^ between molecular and compositional changes to clarify these underlying mechanisms.

Such spatiotemporal studies will be particularly intriguing in light of the similar changes in gene expression and cell viability observed across both compressed regions and opposing tensed areas, suggesting that by day 14, both sides of asymmetrically loaded discs experienced the strain. This contrasts with previous studies that reported distinct cellular responses between the flexed and tensed sides.^25^, ^41^ However, these studies applied static loading, which may induce different long‐term effects on disc biology. Alternatively, asymmetric rotational loading, as applied in this study, may depressurize the NP and impose additional radial stress on the inner AF, thereby modifying the cell's perception of strain in both regions of the disc.^32^

Our research also opens new avenues for exploring the specific contributions of different motions to disc degeneration. Notably, excluding torsion at higher angles of rotation improved cell viability and moderately preserved collagen type II in the AF. This aligns with earlier findings that torsion combined with flexion forces the annular fibers to bear a greater portion of the load, increasing stress and damage near the TZ.^31^ However, GAG levels were similarly reduced in both the group subjected to only extension and bending and the group that included torsion, suggesting that bending may play a more critical role in GAG depletion than torsion. Despite a similar trend of GAG loss from tissue, total GAG release into the medium diverged for these two groups on loading days 7 and 14, indicating a positive accumulation for the torsion‐free group and a negative trend for the group that includes torsion. This suggests potential regional differences within the disc organ and highlights, as for the presence of cracks, the need for a more comprehensive histological or radiological analysis at the whole tissue level. Additionally, degradome studies are needed to clarify if the two combinations of loading lead over time to different GAG degradation products, which fail to be detected with the colorimetric method used in this study.

Discs with less matrix disruption exhibited reduced collagen type I levels in the NP, while those with more significant matrix disruption showed similar or higher collagen type I levels, suggesting that collagen type I may play a compensatory role in response to matrix loss in the translational NP region adjacent to the inner AF. Finally, our analysis of the proinflammatory response to culture under multiaxial loading revealed a strong link with IL‐6, which was, in most groups, upregulated in the outer AF and showed faster accumulation in the medium in groups loaded at higher angles of rotation. The low‐angle extension/bending/torsion exhibited a less prominent IL‐6 release, which may be linked to the reduced number of viable cells observed in this group, possibly affecting the cytokine release. IL‐6 levels have previously been correlated with the extent of tissue damage.^49^ Accordingly, moderate IL‐6 release may reflect the relatively mild GAG loss in this group compared to others. While the association between disc‐related pathologies and IL‐6 has been studied in human serum samples,^50^, ^51^ the specific mechanisms underlying IL‐6 activation in response to disc injury remain unclear and merit further investigation.

In conclusion, the integration of complex motions into ex vivo IVD disease models has highlighted the TZ as a critical initiation region for degeneration, alongside the central NP, which has traditionally been considered the primary site of IVD pathology. The young age of calves used in this study may have influenced the outcomes related to degenerative changes, considering their biological responses can differ from those of older cows. The lengthy whole‐organ preparation required for multiaxial loading, coupled with the limited availability of bioreactors, constrains the number of experimental groups that can be tested simultaneously. The observed compositional changes also necessitate whole‐organ investigation, further restricting the number of possible outcome measures. Future studies of axial rotations will also require establishing parameters for a physiological control group and incorporating static compression as a continuous load on the spine.

Despite these challenges, the outcomes of this study reveal collagen type II, GAG, and their degradation products as potential initiators of degenerative processes, presenting new perspectives for the design and targeted delivery of therapeutics aimed at reinforcing the critical tissue regions or mitigating their breakdown. The development of advanced bioreactors capable of simulating multiaxial loading under controlled biological environments represents a significant advancement in IVD research, bridging the gap between basic and applied science. This innovation promises to deepen our understanding of disc degeneration under various spinal motion conditions and may guide preventative measures or therapies aimed at preserving spine health, particularly in the context of sports and physical activity.

## AUTHOR CONTRIBUTIONS

**A.Š**.: conceptualization, methodology, validation, formal analysis, investigation, visualization, writing‐original draft, writing‐review and editing. **A.R**.: conceptualization, methodology. **F.C**.: conceptualization, methodology, software, **S.H**.: resources, funding acquisition. **M.A**.: conceptualization, funding acquisition. **G.W**.: resources, funding acquisition. **D.L**.: conceptualization, methodology, resources, validation, project administration. **S.J.F**.: conceptualization, funding acquisition.**S.G**.: conceptualization, resources, funding acquisition.

## FUNDING INFORMATION

This work was supported by the Swiss National Science under grant number 189915, the AO Foundation, and AO Spine.

## CONFLICT OF INTEREST STATEMENT

All authors declare that they have no conflicts of interest with respect to this work.

## Supporting information


**Supp. Figure 1.** Changes in cell organization within the central nucleus pulposus, visualized with safranin‐O/fast green staining. Discs subjected to varying loading conditions exhibited cell clustering, in contrast to the single‐cell distribution observed in healthy controls.

**Supp. Figure 2.** Structural and compositional changes in a disc specimen subjected to high‐angle extension, bending and torsion, visualized using different staining methods and immunohistochemistry. (A; arrowhead) Disrupted lamellar organization in the annulus fibrosus (AF) at the transition to nucleus pulposus (NP), further evident in the atypical wavy arrangement of glycosaminoglycans (B) and collagen type II (C). (A, D; asterisks) An exceptionally high content of fibrous collagen type I in the NP.

**Supp. Table 1.** Custom‐designed bovine primer sequences (forward [F], reverse [R], probe [P]) with 5’ FAM and 3′ TAMRA modification, and bovine gene expression assays of commercially available primers/probes.

## Data Availability

The data that supports the findings of this study are available in the supplementary material of this article.
