# Supplementary material for: Multiaxial rotational loading compromises the transition zone of the intervertebral disc: Ex vivo study using next‐generation bioreactors
Source: Bioeng Transl Med. 2025 Jun 8;10(4):e70033. doi: 10.1002/btm2.70033 (PMC12284434; doi:10.1002/btm2.70033)
Supplement: Supplementary file 1 — Supp. Figure 1. Changes in cell organization within the central nucleus pulposus, visualized with safranin‐O/fast green staining. Discs subjected to varying loading conditions exhibited cell clustering, in contrast to the single‐cell distribution observed in healthy controls. [file BTM2-10-e70033-s001.docx]

***Supp. Fig. 1.*** Changes in cell organization within the central nucleus pulposus, visualized with safranin-O/fast green staining. Discs subjected to varying loading conditions exhibited cell clustering, in contrast to the single-cell distribution observed in healthy controls.
