# Supplementary material for: Multiaxial rotational loading compromises the transition zone of the intervertebral disc: Ex vivo study using next‐generation bioreactors
Source: Bioeng Transl Med. 2025 Jun 8;10(4):e70033. doi: 10.1002/btm2.70033 (PMC12284434; doi:10.1002/btm2.70033)
Supplement: Supplementary file 2 — Supp. Figure 2. Structural and compositional changes in a disc specimen subjected to high‐angle extension, bending and torsion, visualized using different staining methods and immunohistochemistry. (A; arrowhead) Disrupted lamellar organization in the annulus fibrosus (AF) at the transition to nucleus pulposus (NP), further evident in the atypical wavy arrangement of glycosaminoglycans (B) and collagen type II (C). (A, D; asterisks) An exceptionally high content of fibrous collagen type I in the NP. [file BTM2-10-e70033-s002.docx]

***Supp. Fig. 2.*** Structural and compositional changes in a disc specimen subjected to high-angle extension, bending and torsion, visualized using different staining methods and immunohistochemistry. (A; arrowhead) Disrupted lamellar organization in the annulus fibrosus (AF) at the transition to nucleus pulposus (NP), further evident in the atypical wavy arrangement of glycosaminoglycans (B) and collagen type II (C). (A, D; asterisks) An exceptionally high content of fibrous collagen type I in the NP.
